# Inflammation-induced endothelial cell activation and angiogenic sprouting are downmodulated by ubiquitin-specific peptidase 20

**DOI:** 10.1101/2025.05.20.655129

**Published:** 2025-05-24

**Authors:** Bipradas Roy, Jiao-hui Wu, Neil J. Freedman, Sudha K. Shenoy

**Affiliations:** 1 Division of Cardiology, Department of Medicine, Duke University Medical Center, Durham, NC 27710, USA; 2 Department of Cell Biology, Duke University Medical Center, Durham, NC 27710, USA

## Abstract

Nuclear factor-κB (NFκB) mediates inflammation-driven angiogenesis, which promotes growth of atherosclerotic plaques and tumors. The deubiquitinase ubiquitin-specific peptidase 20 (USP20) suppresses NFκB activation in vascular smooth muscle cells (SMCs) and attenuates atherosclerosis, but the role of USP20 in endothelial cells (ECs) was undefined. We tested whether USP20 activity diminishes NFκB signaling in ECs and thereby diminishes angiogenesis. Cytokine-induced NFκB activity was elevated in primary ECs isolated from *Usp20*^*−/−*^ mice as compared with ECs from wild-type (WT) mice. Similarly, cytokine-induced NFκB activity was elevated in mouse coronary endothelial cells (MCECs) expressing catalytically inactive USP20 (USP20-DN) or phospho-mimetic USP20(S334D). In contrast, cytokine stimulation of MCECs expressing WT USP20 (USP20-WT) or phospho-resistant USP20(S334A) produced blunted NFκB activity. Assessed by scratch-wound healing and spheroid assays, migration and angiogenesis of MCECs, respectively, were (a) increased by USP20-DN or USP20(S334D), and (b) decreased by USP20-WT or USP20(S334A). Angiogenesis assessed by the aortic ring assay was significantly increased in *Usp20*^*−/−*^ mice and was suppressed by TPCA-1, an inhibitor of NFκB signaling. Angiogenesis was augmented in USP20(S334D) mouse aortic rings but reduced in USP20(S334A) mice. By screening known angiogenesis factors, we identified matrix metalloproteinase 3 (MMP3), a transcriptional target of NFκB, as a gene that is also regulated by USP20 expression and Ser334 phosphorylation. Inhibiting MMP3 reduced angiogenic sprouting in the *Usp20*^*−/−*^ mouse aortic rings. We conclude that USP20 expression inversely correlates with the extent of angiogenesis, and that inhibiting USP20 Ser334 phosphorylation could be a useful strategy to constrain inflammation-driven angiogenesis under pathological circumstances, like cancer and atherosclerosis.

## INTRODUCTION

Cytokine-induced activation of the proinflammatory transcription factor NFκB plays an important role in various pathophysiological conditions, including modulation of angiogenic factors^1,2^. The activation of NFκB signaling depends on IκB kinase-β activation which is modulated by a series of lysine-63-linked, reversible polyubiquitination^3,4^. Activation of proinflammatory cytokine receptors, including tumor necrosis factor receptor (TNFR), Toll-like receptor-4 (TLR4) and interleukin-1 receptor (IL-1R) with their specific ligands, triggers lysine-63-linked ubiquitination of the downstream adaptor TNFR-associated factor (TRAF)^4^. As part of its activation process TRAF autoubiquitinates and subsequently ubiquitinates NFκB essential modulator (NEMO) and TAB2 subunit of the TAK1 kinase complex. Ubiquitinated TAK1 phosphorylates and activates inhibitory κB kinase-β (IKKβ), which in turn phosphorylates not only the p65 subunit of NFκB, but also the inhibitory κBα (IKBα) leading to IκBα degradation, all of which collectively provoke the nuclear translocation of p65 and p50 subunits of NFκB and gene transcription. However, deubiquitinases such as the ubiquitin-specific peptidase 20 (USP20) play a critical role in deubiquitinating TRAF6, resulting in a pause in the NFκB activation cascade and subsequent downregulation of NFκB-mediated transcription of target genes^5^. Vascular smooth muscle cell-specific expression of dominant-negative-USP20 (USP20-DN) increases vascular inflammation, NFκB activation, and atherosclerotic lesions in *Ldlr*^−/−^ mice^6^. The phosphorylation status of USP20 on Ser334 regulates the extrinsic activity of USP20^7^. CRISPR/Cas9-mediated gene-edited phosphorylation-restricted USP20 (USP20-S334A) mice develop less neointimal hyperplasia and reduced VSMC-specific NFκB activation, VCAM expression, and SMC proliferation in mice with carotid endothelial denudation^7^.

NFκB signaling plays a critical role in promoting inflammation-induced angiogenesis in cancer progression^2,8^. Inhibition of NFκB signaling by overexpression of a dominant-negative IκBα significantly decreased in vitro angiogenesis and in vivo tumor vascularity derived from BCPAP cells^8^. NFκB expression in clear cell renal cell carcinoma tissues positively correlates with angiogenic markers, including VEGF and EGFR^9^. Suppression of NFκB activity attenuated angiogenesis in pancreatic cancer cells^10–12^. Because NFκB activity is implicated in angiogenic growth, which can nucleate from endothelial cells, and because USP20 activity can attenuate NFκB signaling, we tested the hypothesis that USP20 regulates EC inflammation and angiogenic growth. Here we summarize our findings from signaling assays that define the role of EC USP20 in NFκB activation and from a core set of angiogenesis assays that reveal the significance of USP20 expression and S334 phosphorylation in this process.

## METHODS

### Materials

Murine IL-1β was purchased from PeproTech. Recombinant mouse VEGF (Cat. No. 493-MV) from R&D Systems; Methyl cellulose (Cat. No. M7027), and Rat collagen type I (Cat. No. 08–115) from Millipore Sigma; Opti-MEM (Cat. No. 51985–034) from Thermo Fisher Scientific; Antibodies used for immunoblotting and immunofluorescence are listed below, along with their sources. All antibodies were diluted at 1:1000 for immunoblotting and 1:100 for immunofluorescence, unless stated otherwise. Rabbit monoclonal anti-phospho-p65(Ser536) (Cat. No. 3033, 1:5000), and rabbit monoclonal anti-GAPDH conjugated to HRP (Cat. No. 3683S, 1:5000) from Cell Signaling Technologies; mouse monoclonal anti-β-actin (Cat. No. A5441, 1:10,000) from Sigma-Aldrich; mouse monoclonal anti-CD31 (Cat. No. MA5–13188), and Alexa Fluor 594-conjugated wheat germ agglutinin (WGA; Cat. No. W11262) from Thermo Fisher Scientific; Horseradish peroxidase-conjugated secondary antibodies were from Cell Signaling Technology, Rockland Immunochemicals and Bethyl Laboratories, Inc; they were used at a dilution of 1:5000. Secondary antibodies conjugated to Alexa Fluor 488^®^ and Alexa Fluor 546^®^ were obtained from Invitrogen; they were used at 1:100. MMP-3 inhibitor II (Cat. No. 444225) was from Millipore Sigma.

### Mice

All animal experiments were conducted in accordance to protocols that were approved by the Duke University Institutional Animal Care and Use Committee. From the Sanger Institute, we obtained *Usp20* mice with a genetrap null allele (Usp20^tm1a^(EUCOMM)). Mating this with Flp-transgenic mice removed the lacZ and neo cassettes of the “knockout-first” allele,^13^ thereby restoring *Usp20* expression and rendering the *Usp20* allele “floxed” (loxP sites flank exons 7 and 8). By breeding *Usp20*^*flox/flox*^ with CMV-Cre mice, we obtained *Usp20*^*−/−*^ (USP20-KO) mice, which are born at expected rates and are phenotypically normal under non-stressed conditions. *Usp20*^*−/−*^ were back-crossed into C57BL/6J, and heterozygous *Usp20*−/+ were bred to obtain *Usp20*^+/+^ wild type (WT) and *Usp20*^−/−^ littermates that were used in this study (Figure S1). USP20-S334D and USP20-S334A [6] mice were generated by the CRISPR/Cas9-mediated gene editing with the help of Duke Transgenic Core facility (Figure S2).

### Cell lines

Aortic ECs were isolated from the descending thoracic aortas of *Usp20*^*−/−*^, and WT that were matched by age and sex. For a single line of primary ECs, aortas from 2–3 mice per genotype were isolated and cultured as reported previously^14^. The MCEC (mouse cardiac endothelial cell) line was obtained from Cedarlane. The MCECs were grown in 150 mm cell culture plates containing Dulbecco’s Modified Eagle Medium (DMEM) supplemented with 10% fetal bovine serum (FBS) (v/v) and 1% (v/v) penicillin/streptomycin (P/S). 0.05% trypsin/EDTA (GE life Sciences) was used to culture the cells.

### Adenoviral transduction

Recombinant adenovirus (AV) expressing HA-tagged USP20 WT (USP20-WT), USP20 dominant-negative (USP20-DN), USP20 phospho-mimetic (USP20-S334D), or USP20 phospho-resistant (USP20-S334A) mutants and eGFP (control) were generated in our lab according to our previously published protocols^6,7,15^. MCECs were incubated with low serum (0.2%) media for 6 hours following transduction with an equivalent MOI of recombinant adenoviruses for 9 hours and serum-starved for 2 hours before the individual assays, including WB, spheroid-based sprouting, and scratch wound healing migration assays. In parallel, the transduction efficiency of the adenoviruses was evaluated by GFP fluorescence and HA epitope immunostaining, and subsequent confocal microscopy followed by cell count analyses (Figure S3).

### Proinflammatory cytokine stimulation

MCECs that were transduced with recombinant adenovirus were stimulated with 10 ng/μl IL-1β for 15 minutes, after which the total protein was extracted with 2 × SDS-PAGE protein sample buffer. The mouse aortic primary ECs were serum-starved overnight and subsequently stimulated with 10 ng/μl IL-1β for 5, 10, 15, 30, and 60 minutes before protein extraction with 2 × SDS-PAGE protein sample buffer.

### Aortic ring sprouting assay

The aortic ring sprouting assay was performed according to the protocol mentioned elsewhere^16^. Briefly, aortas were isolated from the age and sex matched WT, USP20^−/−^, USP20-S334A, and USP20-S334D male and female mice. The isolated aorta was immediately washed twice with DPBS containing calcium and magnesium to remove the residual blood and transferred to a 100 mm plate containing complete DMEM supplemented with 10% FBS and 1% P/S. Under a stereo microscope, the residual fat from the aorta was removed with the help of a sterilized tweezer. Additionally, the side branches of the aorta were removed with the help of a sharp and sterilized scalpel. The cleaned aorta was immediately transferred to a 100 mm plate containing complete DMEM supplemented with 10% FBS and 1% P/S. The cleaned aorta was dissected into small ring-like fragments with an approximate height of 0.8 mm. From a single aorta, 42–48 aortic rings were made. The aortic rings were transferred to a 6-well plate containing DMEM supplemented with 0.2% FBS and 1% P/S and incubated overnight in a CO_2_ incubator. The next day, a collagen stock solution was prepared in a 50 ml tube by adding 1 mg/ml collagen in ice-cold Opti-MEM supplemented with 10% FBS and 1% P/S, and the stock solution was maintained in ice. 200 μl of the ice-cold collagen stock solution was pipetted into each well of a 24-well plate, and immediately, 7 aortic rings were planted in each well on top of the collagen matrix with the help of a sharp-tipped sterilized tweezer. The aortic rings were planted on the collagen matrix in such a way that the luminal axis of each aortic ring was perpendicular to the bottom of the wall. The plate was then incubated in a CO_2_ incubator for 1 hour, and subsequently, 250 μl of Opti-MEM supplemented with 10% FBS, 1% P/S, and 30 ng/ml VEGF was added in each well of the 24-well plate containing the collagen matrix with aortic rings embedded in it. At this stage, the desired drug, for example, TPCA-1, was added to the Opti-MEM to study its role in aortic ring sprouting. The plate was then incubated in a CO_2_ incubator for 5 days, and then images were captured by a Zeiss Axio Observer Z1 microscope with 5x magnification. The relative sprouting area of each aortic ring captured under high power field (HPF) was subsequently measured with the NIH ImageJ software.

#### Proteome profiler analyses:

The expression levels of angiogenesis-related proteins were determined with the proteome profiler mouse angiogenesis array kit according to the manufacturer’s protocol. (Proteome Profiler Mouse Angiogenesis Array Kit, Cat. No. ARY015, Briefly, the Proteome Profiler Mouse Angiogenesis Array Kit utilizes a membrane-based sandwich immunoassay. Initially, samples are combined with a mixture of biotinylated detection antibodies (Step 1), followed by incubation with the array membrane that has capture antibodies for specific target proteins printed in duplicate (Step 2). The captured proteins are then visualized using chemiluminescent detection reagents (Step 3). The intensity of the signal generated correlates with the amount of bound analyte. Analytes include components of angiogenesis pathway and include soluble growth factors, differentiation factors, extracellular matrix components, proteases, membrane-bound receptors, and intracellular signaling molecules.

To extract proteins from the sprouted aortic rings, the wells of the 24-well plates containing the aortic rings were washed twice with PBS (with Ca^2+^ & Mg^2+^), and 150μl RIPA buffer^6^ was added in each well of the plates. The plates were incubated on a shaker overnight at 4°C. Supernatants were collected from the wells, and protein concentrations were measured with the BCA protein assay kit. Equivalent protein amounts of experimental samples were used for the angiogenesis proteome profiling assay.

### Immunofluorescence staining and confocal imaging

Immunofluorescence staining and confocal imaging of the cultured MCECs was performed according to our previously published protocol^6^. Briefly, MCECs at their 50–60% confluency was transduced with adenovirus expressing HA-tagged USP20-WT, USP20-DN, USP20-S333A, USP20-S333D, or GFP for 12 hours. After the transduction, MCECs were incubated with the fresh complete DMEM supplemented with 10% FBS and 1 % P/S for 12 hours. Then the MCECs were seeded on polyD-lysine-coated 35-mm glass bottom plates. 48 hours post-transduction MCECs were fixed with 5% formaldehyde diluted in Dulbecco’s PBS (DPBS) without calcium and magnesium, and subsequently washed three times with DPBS. The fixed MCECs were permeabilized with 0.01% Triton X-100 in DPBS containing 2% BSA for 30 mins and incubated with the appropriate primary antibody overnight at 4 °C. The next day, MCECs were washed with DPBS three times and incubated with the cognate secondary antibody as well as DAPI in a dark chamber for 1 hour at room temperature (RT). The stained MCECs were visualized with Zeiss LSM-510 META confocal microscope using a 63x oil immersion objective.

For the immunofluorescence staining of sprouted aortic rings cultured in a collagen matrix, they were fixed with 5% formaldehyde diluted in DPBS without calcium and magnesium for 40 mins at RT and subsequently washed three times with DPBS. The fixed aortic rings were permeabilized with 0.01% Triton X-100 in DPBS containing 2% BSA for 1 hour and incubated with mouse anti-CD31 IgG overnight at 4 °C. Aortic rings were washed three times with DPBS and incubated with Alexa Fluor 488 conjugated cognate secondary antibody, DAPI and Alexa Fluor 594 conjugated Wheat Germ Agglutinin (WGA, catalog no. W11262, Thermo Fisher Scientific) in a dark chamber for 1 hour at room temperature (RT). The images of the sprouted aortic rings were captured with a Zeiss LSM 510 META confocal microscope.

### Spheroid-based sprouting Assay

For the spheroid-based sprouting assay 80,000 MCECs were diluted in 4 mL of DMEM supplemented with 10% FBS, 1% P/S, and 20% Methocel. The MCECs suspension was transferred to a sterile multichannel pipette reservoir and immediately 25 μL of MCECs suspension was pipetted as drops onto a 150 mm cell culture plate with the help of a multichannel pipette. There were approximately 400 cells in each 25 uL drop. The plate was incubated upside down in a CO_2_ incubator for 24 hours for spheroid formation. The hanging drops containing the spheroids were washed off with 10 mL PBS by pipetting them using a 10 mL serological pipette. The spheroid suspension was immediately transferred to a 15 mL conical tube and centrifuged at 200 g for 5 min. The supernatant was aspirated very carefully and 2 mL ice-cold Methocel supplemented with 20% FBS was added into the tube. Separately, a 4 mL collagen stock solution was prepared by adding 1 mg/mL collagen in ice-cold DMEM supplemented with 10% FBS and 1% P/S. The pH of the collagen stock solution was adjusted by adding 2.5 μL of 10 N NaOH which was visually confirmed with the color change from yellow to orange. The ice-cold Methocel containing the spheroids was mixed with the collagen stock solution, and immediately, 250 μL of the spheroid suspension was pipetted in each well of a 24-well cell culture plate. The plate was incubated in a CO_2_ incubator for 1 hour to polymerize and solidify the collagen. 250 μL of DMEM supplemented with 10% FBS, 1% P/S, and 30 ng/mL VEGF was added in each well of the 24-well plate containing the collagen matrix with spheroids embedded in it. If needed, inhibitors such as TPCA were added at this step. The plate was then incubated in a CO_2_ incubator for 48 hours. The images of sprout growth from the spheroids embedded in the collagen matrix were captured by a Zeiss Axio Observer Z1 microscope with 10x magnification. The number of nodes in a sprouted spheroid were quantified with the NIH ImageJ software.

### Scratch wound healing migration assay

MCECs were grown to form a confluent (~90%) monolayer in 6-well plates containing DMEM supplemented with 10% FBS and 1% P/S. The MCECs monolayer was scratched with a 1 mL pipette tip to make wounds, and immediately, the medium was aspirated to remove the floating cells detached from the wounds of the MCECs monolayer. The plates were washed once with DPBS, and 2 mL fresh DMEM supplemented with 10% FBS and 1% P/S was added in each well of the 6-well plates. If needed, inhibitors such as TPCA were added at this step. The images of the wounds were captured at different time points (0 h, 8 h, and 16h) by a Zeiss Axio Observer Z1 microscope with 2.5x magnification. At least 10 images were taken from each of the experimental groups. The percentage of gap closure from each image was measured by the wound width using the ImageJ software. The migration rate of MCECs was calculated by the formula: (width at 0 h – width at indicated time point) × 100% / width at 0 h^17^.

### Western blotting

To determine the protein levels of phos-p65 in IL-1β-stimulated MCECs or mouse aortic primary ECs, immunoblots were performed using the total protein extracted in 2 × SDS-PAGE protein sample buffer. Specific protein bands were separated by 10% SDS-PAGE gels and subsequently transferred to nitrocellulose membranes. The membranes were blocked with 5% (w/v) dried skim milk in TTBS buffer [0.2% (v/v) Tween 20, 10 mM Tris-Cl (pH 8.0 at 25 °C), 150 mM NaCl]. The blocked membranes were incubated with phos-p65, β-actin or GAPDH primary antibodies overnight at 4 °C. Membranes were washed three times with TTBS and incubated with the appropriate secondary antibodies for 1 hour at room temperature. Protein bands were detected with enhanced chemiluminescence (SuperSignal West Pico Reagent, Pierce) using a charge-coupled device camera system (Bio-Rad Chemidoc-XRS). Densitometry of protein bands was performed with Image Lab software (Bio-Rad).

### Statistical Analyses

All assays were repeated at least three independent times, and the data from all experiments were averaged and presented as means ± SD. A student t-test was performed to determine statistical significance between two groups, and one-way/two-way ANOVA followed by Holm-Šídák’s post hoc test for multiple comparisons using the GraphPad Prism 10 software (GraphPad, Inc). The level of statistical significance was set at p < 0.05.

## RESULTS

### Proinflammatory cytokine-induced NFκB activity is elevated in primary ECs lacking USP20

**Figure 1.**
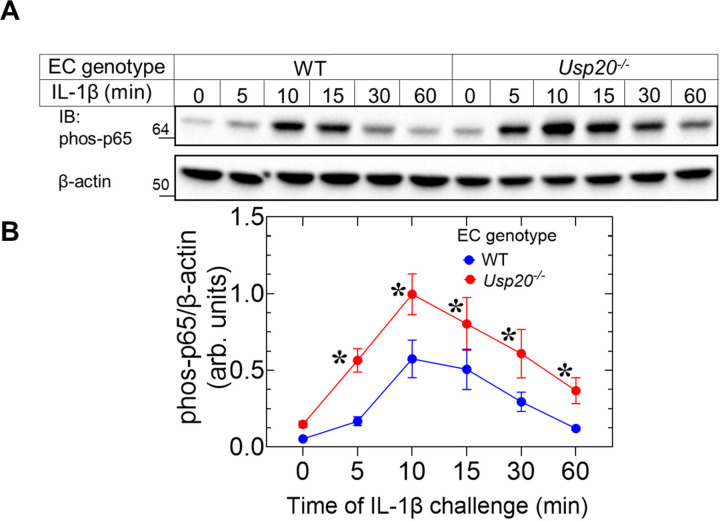
Proinflammatory cytokine stimulation augments NFκB activation in aortic primary ECs lacking USP20 (A) Representative WB bands of IL-1β (10 ng/ml)-induced phosphor-p65 protein levels at different time points in aortic primary ECs obtained from WT and *Usp20*^*−/−*^ mice. (B) Quantification of the WB bands shown in panel A. N=6 in each group. Two-way ANOVA followed by Holm-Šídák’s multiple comparisons tests were performed to determine the statistical significance. *p < 0.01 vs WT.

Our previous *in vitro* and *ex vivo* studies demonstrated that proinflammatory stimuli (e.g., LPS, IL-1β and TNF) increased the NFκB activity in mouse SMCs lacking USP20^5,6^. However, the role of USP20 in proinflammatory cytokine-induced NFκB activity in ECs was undefined. Therefore, we isolated aortic primary ECs from *Usp20*^*−/−*^ mice to test if USP20 expression affects cytokine stimulated inflammatory signaling. 10 ng/mL IL-1β stimulation of ECs significantly increased the phospho-p65 protein levels in *Usp20*^*−/−*^ cells at different time points as compared with ECs isolated from WT littermate controls (Fig. 1, A & B). Accordingly, USP20 regulates NFκB activity in SMCs as well as ECs.

### USP20 Serine334 phosphorylation increases NFκB signaling in ECs.

**Figure 2.**
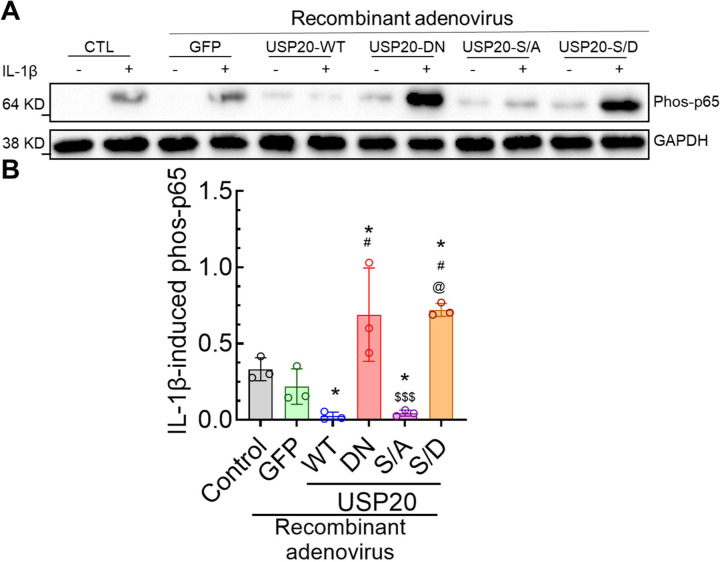
Phosphorylation of USP20 on Ser334 augments NFκB activity in proinflammatory cytokine-stimulated ECs (A) Representative WB data of IL-1β-induced phos-p65 protein levels in cultured MCECs transduced with recombinant adenoviruses encoding N-terminal HA-tagged USP20 constructs expressing USP20-WT, USP20-DN, USP20-S334A, and USP20-S334D or no virus (Control, CTL). N=3 in each group. One-way ANOVA followed by Holm-Šídák’s multiple comparisons tests were performed to determine the statistical significance. *p < 0.05 vs control, ^#^*p* < 0.01 vs USP20-WT, ^$ $ $^*p* < 0.01 vs USP20-DN, ^@^*p* < 0.01 vs USP20-S/A.

The binding of USP20 with its substrate promotes deubiquitinase activity, and this activity is regulated by the phosphorylation status of Ser334 or Ser333 in murine and human USP20, respectively^6,15,18^. Our previous study demonstrated that non-phosphorylated USP20 (USP20-S334A) significantly increased USP20 activity and attenuated NFκB activation compared with WT control^7^. However, the role of the phosphorylation status of USP20 in proinflammatory cytokine-induced NFκB activation in ECs was not tested. We therefore evaluated the effect of USP20 (USP20-WT), inactive USP20 (USP20-dominant negative or USP20-DN), phospho-resistant USP20 (USP20-S334A), and phospho-mimetic USP20 (USP20-S334D) on NFκB activation triggered by IL-1β. We detected significantly more NFκB activity in cells expressing USP20-DN and USP20-S334D than in cells expressing USP20, USP20-S334A, and GFP (Figure 2A–B). Additionally, expression of WT and S334A constructs significantly reduced NFκB activation compared to cells expressing GFP. In our assays, we also used cells that were not transduced with adenovirus, in which NFκB activity was similar to that obtained with GFP expression (Fig. 2, A–B). Accordingly, we conclude that USP20 activity in ECs negatively regulates NFκB signaling, whereas USP20 S334 phosphorylation promotes NFκB signaling.

### USP20 activity and de-phosphorylation at Serine334 diminish migration of endothelial cells

**Figure 3.**
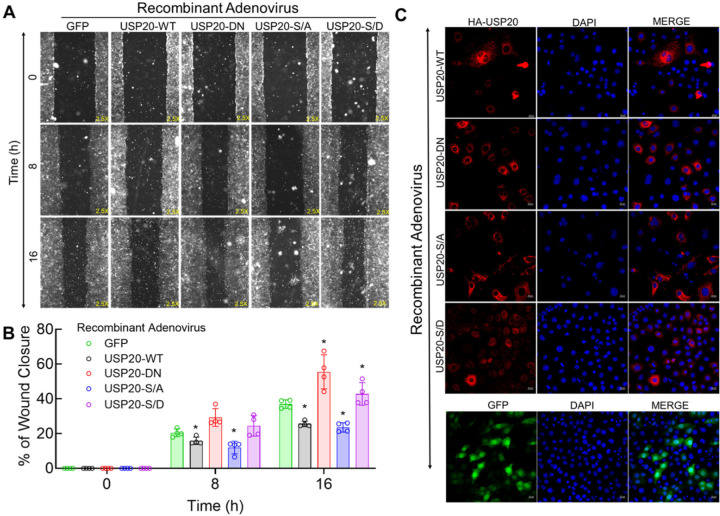
Effect of USP20 activity and Ser334 phosphorylation on endothelial cell migration. (A) Representative micrographs of scratch-wound healing assay at different time points (0, 8 & 16 hours) using MCECs transduced with recombinant adenoviruses encoding N-terminal HA-tagged USP20 constructs that were USP20-WT, USP20-DN, USP20-S334A, and USP20-S334D. (B) Quantification of the scratch-wound healing assay stated in panel A. N=4 in each condition, 6–11 micrographs were quantified for each condition. Two-way-ANOVA followed by Holm-Šídák’s multiple comparisons tests were performed to determine the statistical significance. *p < 0.05 vs GFP as indicated. (C) Immunofluorescence images of MCECs transduced with recombinant adenoviruses encoding N-terminal HA-tagged USP20 constructs.

To test the role of USP20 and its phosphorylation status in EC migration, we performed the scratch-wound healing assay using MCECs transduced with recombinant AVs, encoding GFP, USP20-WT, USP20-DN, USP20-S334A, and USP20-S334D. MCEC migration was significantly increased in cells expressing USP20-DN compared with cells expressing control GFP protein (*p* < 0.05) (Fig. 3, A and B). Likewise, MCEC migration was significantly increased by USP20 S334D compared with the GFP controls (*p* < 0.05 after 16 hours) (Fig. 3, A and B). Whereas MCEC migration was significantly decreased by USP20-WT compared with the GFP controls (*p* < 0.05 after 16 hours) (Fig. 3, A and B). Similarly, MCEC migration was significantly decreased by USP20-S334A compared with the GFP controls (*p* < 0.05 after 8 hours and *p* < 0.0001 after 16 hours) (Fig. 3, A and B). To evaluate the transduction efficiency of the recombinant adenoviruses, we performed immunostaining of the transduced MCEC for HA-USP20 (red) and DAPI (blue) (Fig. 3, C). The transduction efficiency of all the recombinant adenoviruses was comparable, with the exception of USP20-WT, which was at <50% for bright cells (Figure S3).

### The angiogenic sprouting of MCECs is diminished by the expression of WT and phospho-resistant USP20-S334A, while it is exacerbated by USP20-DN and phospho-mimetic USP20-S334D.

**Figure 4.**
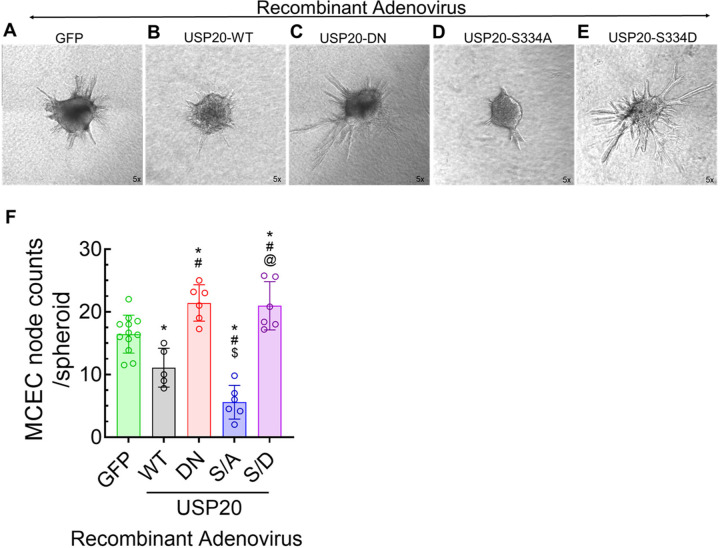
Effect of USP20 and Ser334 phosphorylation in modulating sprouting of MCEC spheroids. (A) Representative photomicrographs of the spheroid assay using MCECs transfected with GFP. Magnification 5x. (B-E) Representative micrographs of spheroid sprouting in MCECs transduced with recombinant adenoviruses encoding N-terminal HA-tagged USP20 constructs: USP20-WT, USP20-DN, USP20-S334A, and USP20-S334D. Magnification 5x. (F) Quantification of the spheroid assay shown in panels A-E. N = 5–12 wells, each well represents the average node counts of 1–5 spheroids. Each bar represents the mean ± SD. *p <0.05 as follows:* * vs GFP, ^*#*^ vs USP20-WT, ^*$*^ vs USP20-CH, ^@^ vs USP20-S334A, as indicated. One-way ANOVA followed by Holm-Šídák’s multiple comparisons tests.

The spheroid-based sprouting assay provides a suitable *in vitro* 3D model to study the role of genetic alterations or pharmacological compounds on angiogenic potential of endothelial cells^19^. To test the role of USP20 and its phosphorylation status in EC sprouting, we performed the spheroid-based sprouting assay using MCECs transduced with recombinant adenoviruses, expressing USP20-WT, USP20-DN, USP20-S334A, and USP20-S334D (Fig. 4A–E). While all experimental conditions retained spheroid structures, we found differences in the length and quantity of elongated spikes at the external boundary. We infer these spikes to represent sprouting similar to EC sprouting reported for aorta slices. USP20-WT and USP20-S334A transduced MCECs formed weak or no sprouts from spheroids compared with the GFP-treated MCECs (Fig. 4F), whereas angiogenic sprouting was significantly increased in USP20-DN and USP20-S334D transduced MCECs compared with the GFP-treated MCECs (Fig. 4, F).

### Angiogenic sprouting is increased in USP20 knockout or phospho-mimetic (USP20-S334D) mice and decreased in USP20 phospho-resistant (USP20-S334A) mice

**Figure 5.**
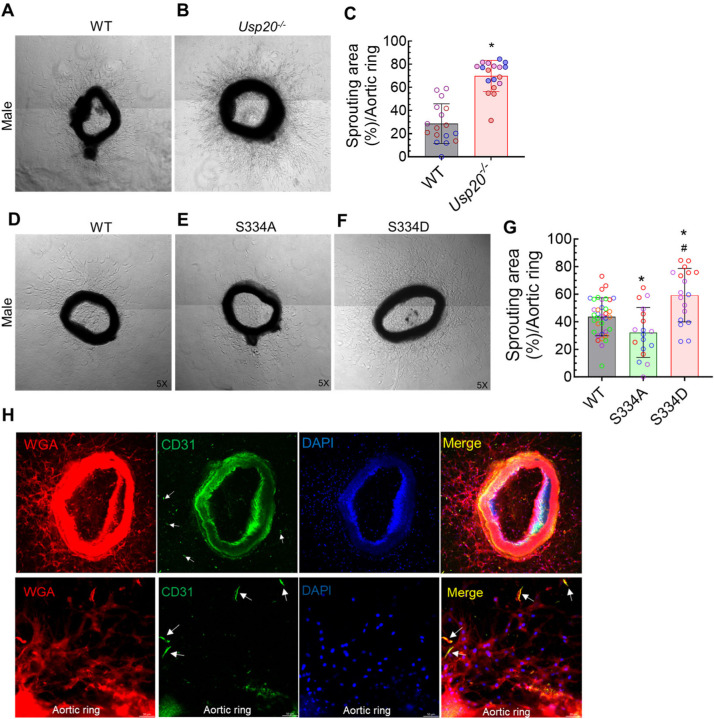
Effect of USP20 and Ser334 phosphorylation on aortic angiogenesis. (A-B) Representative micrographs of aortic sections isolated from 3-month-old WT and *Usp20*^−/−^ male mice. (C) Quantification of the sprouting area of WT and *USsp20*^*−/−*^ samples from A-B. Magnification 5x. N= 3 mice for each group; each mouse represents 6 dots; each dot represents the average sprouting area of 4–7 aortic rings. Each bar summarizes mean ± SD. An unpaired student’s t-test was performed to determine the statistical significance. * *p* < 0.01 vs WT. (D-F) Representative micrographs of the *ex vivo* aortic ring sprouting assay using the aorta isolated from 3-month-old WT, phospho-resistant USP20 (S334A), and phospho-mimetic USP20 (S334D) male mice, respectively. (G) Quantification of the sprouting area stated in panels D-F. Magnification 5x. N= 6 WT, 3 USP20-S334A and 3 S334D mice; each mouse represents 6 dots; each dot represents the average sprouting area of 5–7 aortic rings. Each bar summarizes mean ± SD of quantitation. One-way ANOVA followed by Holm-Šídák’s multiple comparisons tests were performed to determine the statistical significance. *p < 0.05 vs WT-CTL, ^#^*p* < 0.01 vs USP20-S334A as indicated. WT, Wild-Type. (H) Immunofluorescence staining of a sprouted aortic ring (obtained from a *Usp20*^−/−^ male mouse) with WGA (red), CD31 (green), and DAPI (blue) markers. The lower row of images shows an enlarged section of a stained aortic ring. The white arrows in the merged image indicate the presence of endothelial cells (yellow). Scale bar = 50 μm

To recapitulate the findings from our *in vitro* cell culture study in an animal model, we performed the *ex vivo* aortic ring sprouting assay^20^ with the following mice: USP20 knockout (*Usp20*^−/−^), USP20 phospho-resistant (USP20-S334A), USP20 phospho-mimetic (USP20-S334D), and WT control. Aortic ring sprouting was significantly increased in both male and female *Usp20*^*−/−*^ mice as compared with their WT littermates (*p* < 0.05) (Fig. 5, A–C, S3. A-C). Similarly, aortic ring sprouting was significantly increased in the USP20-S334D male and female mice relative to their WT littermates (*p* < 0.05) (Fig. 5, D–G, S3. D-G). However, the aortic ring sprouting was significantly decreased in the USP20-S334A male and female mice relative to their WT littermates (*p* < 0.05) (Fig. 5, D–G, S3, D-G). Although the angiogenic sprouting is predominantly carried out by the ECs, the sprouting ECs closely interact with other cell types, including the pericytes, macrophages, and fibroblasts, in a paracrine manner to facilitate the whole process of angiogenesis^20^. To evaluate the involvement of ECs in the sprouted aortic ring, we performed immunofluorescence staining for the endothelial cell marker CD31 (green). We detected the presence of ECs in the sprouted aortic ring (yellow cells indicated by the white arrows in the merged image) (Fig. 5, H), suggesting that our experimental conditions are conducive to EC activation and angiogenesis.

### Migration and angiogenic sprouting of ECs are promoted by NFκB activity

**Figure 6.**
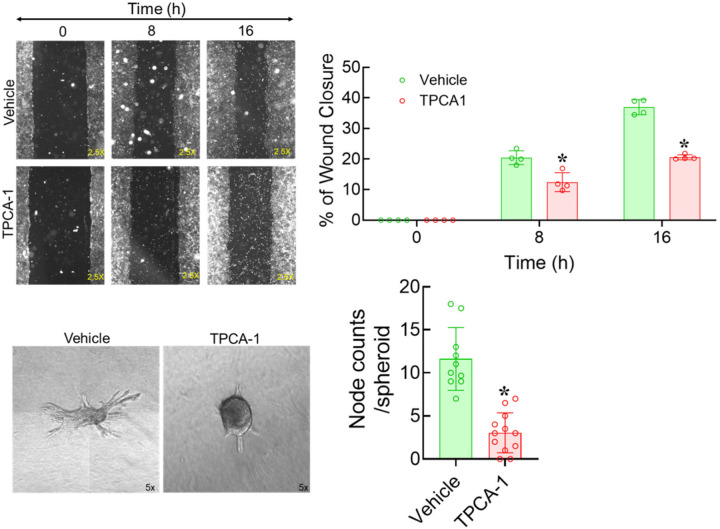
Migration and sprouting of MCECs are blocked by TPCA-1. (A) Representative micrographs of scratch-wound healing assay at different time points (0, 8 & 16 hours) using MCECs treated with vehicle (DMSO) and 10 μM TPCA-1, a potent IKKβ inhibitor. Magnification 2.5x. (B) Quantification of the scratch-wound healing assay stated in panel A. N=4 in each condition, 6–11 micrographs were quantified for each condition. *p < 0.01 vs GFP as indicated, two-way ANOVA followed by Holm-Šídák’s multiple comparison tests were performed to determine statistical significance. (C-D) Representative micrographs of the spheroid assay using MCECs with vehicle, C, and with 10 μM TPCA-1 treatment, D. Magnification 5x. (E) Quantification of the spheroid assay stated in panels C & D. N = 10–12 wells, each well represents the average node counts of 1–3 spheroids. Each bar represents the mean ± SD. A student t-test was performed to determine the statistical significance. *p<0.01 vs control as indicated.

USP20 attenuates EC-specific NFκB activity as revealed by the increased phos-p65 levels in the ECs obtained from *Usp20*^−/−^ mice (Figure 1). Similarly, NFκB activity is elevated in the MCECs transduced with recombinant AVs encoding USP20-DN and USP20-S334D mutants (Figure 2). NFκB signaling has been implicated as a proangiogenic factor in pathological angiogenesis, especially in tumor growth and metastasis^21^. For example, constitutive activation of NFκB is associated with increased angiogenesis in colorectal cancer^22^. To evaluate whether the effect of USP20 on NFκB signaling is correlated with the angiogenic sprouting patterns seen in aortic sections, we conducted targeted inhibition of NFκB in our assays. TPCA-1 is a widely used inhibitor of NFκB signaling that selectively inhibits the upstream kinase IKK-2^23^. Accordingly, we evaluated the effect of TPCA in our scratch wound-healing and spheroid assays by treating MCECs ± 10 μM TPCA-1. Migration of MCECs in the wound scratch assay was significantly reduced by TPCA as compared with untreated MCECs (*p* < 0.01) (Fig. 6, A and B). Similarly, TPCA-1 treatment significantly decreased MCECs sprouting relative to the non-treated controls (*p* < 0.01) (Fig. 6, C–E). Taken together, these results strongly suggest that a reduction in NFκB signaling by USP20 activity can impact EC sprouting in a similar fashion to the reduction of NFκB activity by TPCA-1.

### NFκB inhibitor diminishes sprouting of aortic rings prepared from WT and *Usp20*^*−/−*^ mice.

**Figure 7.**
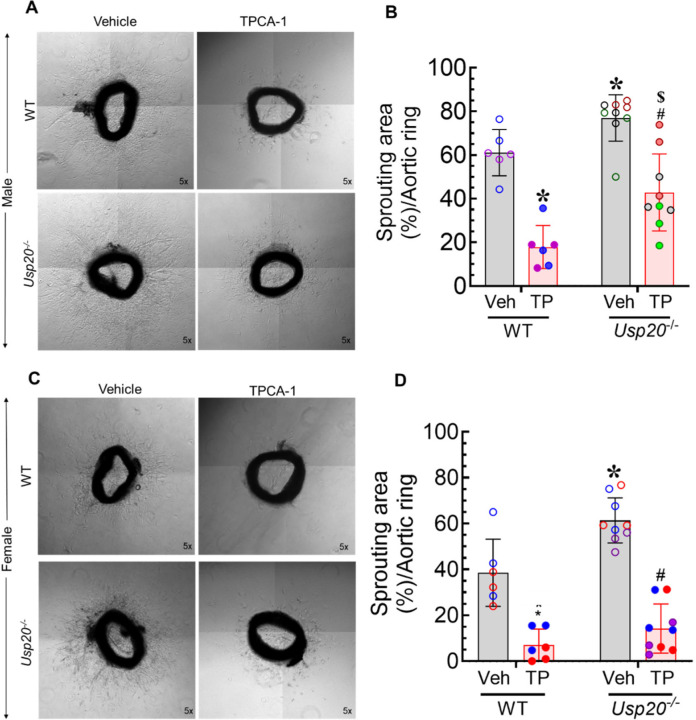
NFκB inhibition decreases aortic angiogenesis. (A) Representative micrographs of the *ex vivo* aortic ring sprouting assay with ±10 μM TPCA-1 treatment using aorta isolated from 3-month-old WT and *Usp20*^−/−^ male mice. (B) Quantification of the sprouting area stated in panel A. Magnification 5x. N = 2 male WT male and 3 male *Usp20*^−/−^ mice; each mouse represents 6 dots (3 untreated, open circles and 3 treated with TPCA-1, filled circles); each dot represents the average sprouting area of 5–7 aortic rings. Each bar represents the mean ± SD. Two-way ANOVA and Holm-Šídák’s multiple comparisons tests were performed to determine the statistical significance. *p < 0.01 vs WT-CTL, ^#^*p* < 0.01 vs *Usp20*^−/−^-CTL, ^$^*p* < 0.01 vs WT-TP as indicated. (C) Representative micrographs of the *ex vivo* aortic ring sprouting assay with 10 μM TPCA-1 treatment using aorta isolated from 3-month-old WT and *Usp20*^−/−^ female mice. (D) Quantification of the sprouting area stated in panel C. Magnification 5x. N = 2 female WT and 3 female *Usp20*^−/−^; each mouse represents 6 dots; each dot represents the average sprouting area of 5–7 aortic rings. Each bar represents mean ± SD. Two-way ANOVA and Holm-Šídák’s multiple comparisons tests were performed to determine the statistical significance. *p < 0.01, and ^#^*p* < 0.01 vs *Usp20*^−/−^-CTL as indicated. CTL, Control; WT, Wild-Type; TP, TPCA1.

NFκB signaling is increased in *Usp20*^−/−^ ECs (Figure 1), and concordantly, aortic sprouting is also increased in *Usp20*^−/−^ (Fig. 7, A–D) as compared with WT conditions. To determine whether the increase in sprouting observed in *Usp20*^−/−^ mice engages NFκB activity, we employed *Usp20*^−/−^ male and female mice to perform the aortic ring sprouting assay ± TPCA-1 treatment. As shown in Figure 7, inhibition of NFκB activity with TPCA-1 significantly decreased aortic ring sprouting in WT and *Usp20*^−/−^ male mice compared with their non-treated controls (*p* < 0.01) (Fig. 7, A, B). Similarly, TPCA-1 treatment significantly decreased the aortic ring sprouting in WT and *Usp20*^−/−^ female mice compared with their non-treated controls (*p* < 0.01) (Fig. 7, C, D). These results suggest that aortic sprouting in both WT and *Usp20*^−/−^ employs NFκB activity.

### Identification of angiogenic factors modulated by USP20 expression and USP20 phosphorylation

**Figure 8.**
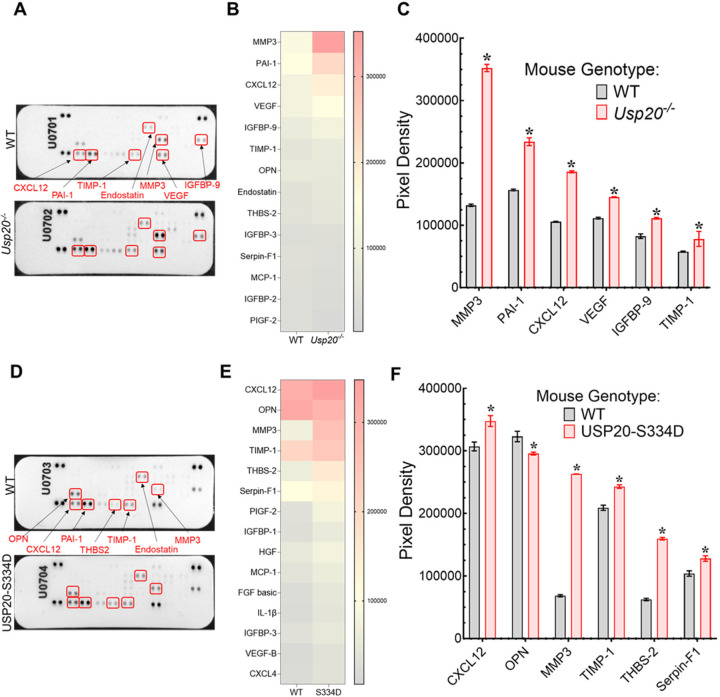
Proteome profiling of angiogenic proteins using WT and *Usp20*^−/−^ aortic sprout protein extracts. (A) Antibody array membranes were incubated with the proteins obtained from WT and *Usp20*^−/−^ mice aortic sprouts. (B) All the 53 angiogenic proteins spotted in panel A were quantified, and the top 15 proteins detected are shown in the heatmap. (C) List of differentially expressed angiogenic proteins elevated in the *Usp20*^−/−^ mouse. Each bar represents the mean ± SD, N=2. Two-way ANOVA and Holm-Šídák’s multiple comparisons tests were performed to determine the statistical significance. *p < 0.01 vs WT. (D) Antibody array membranes were incubated with the proteins obtained from WT and USP20-S334D mice aortic sprouts. (E) All the 53 angiogenic proteins spotted in panel D were quantified, and the top 15 proteins detected are shown in the heatmap. (E) List of differentially expressed angiogenic proteins in the USP20-S334D mouse. Each bar represents the mean ± SD, N=2. Two-way ANOVA followed by Holm-Šídák’s multiple comparisons tests were performed to determine the statistical significance. *p < 0.01 vs WT.

We hypothesized that NFκB signaling promotes gene expression of pro-angiogenic factors, and USP20 activity could regulate the downstream signaling by desensitizing NFκB signaling. To identify specific angiogenic factors that are regulated by USP20, we solubilized aortic sprouts from WT and *Usp20*^−/−^ mice to extract respective total protein samples and then used equivalent amounts of each protein extract to perform a protein profiler assay, which is a membrane-based sandwich immunoassay of an antibody array containing 53 angiogenesis-related capture antibodies (Fig. 8, A, B). We identified six differentially expressed angiogenic proteins, namely (1) matrix metalloproteinases 3 (MMP3), (2) Plasminogen activator inhibitor-1 (PAI-1), (3) C-X-C Motif Chemokine Ligand 12 (CXCL12), (4) vascular endothelial growth factor (VEGF), (5) insulin-like growth factor-binding proteins 9 (IGFBP-9), and (6) Tissue inhibitor of metalloproteinases 1 (TIMP-1) that are significantly increased in the *Usp20*^−/−^ aortic sprouts as compared with WT sprouts (Fig. 8, A–C). Next, we performed the proteome profiler assay using the proteins extracted from sprouted aortic rings of USP20-S334D and congenic WT (Fig. 8, D, E) in order to identify specific angiogenic factors regulated by S334 phosphorylation of USP20. We identified six differentially expressed angiogenic proteins that are significantly altered in the USP20-S334D aortic sprouts as compared with WT sprouts, namely (1) CXCL12, (2) Osteopontin (OPN), (3) MMP3, (4) TIMP-1, (5) Thrombospondin-2 (TSP-2), and (6) Serpin-F1, (Fig. 8, F).

While we detected factors that were uniquely present in S334D versus Usp20−/− samples, the top hits also showed overlap in terms of common angiogeneic factors that were affected. The common factors MMP3, and CXCL12 are regulated by NFκB signaling in different contexts. For instance, NFκB directly regulates MMP3 expression in macrophages in atherosclerotic lesions through binding directly to the MMP3 promoter region with its p50 and p65 subunits^24^. Lymphotoxin-β receptor-induced activation of noncanonical NFκB pathway upregulates CXCL12 expression in HUVECs^25^. On the other hand, both NFκB and TIMP-1 regulate levels of MMPs^26^ although an inverse correlation between MMP and TIMP-1 has also been reported^27^. However, there were no studies reported yet about the role of NFκB-induced modulation of IGFBP-9 or Serpin-F1 in vascular cells.

### Inhibition of MMP-3 decreased angiogenic sprouting in *Usp20*^−/−^ mouse

**Figure 9.**
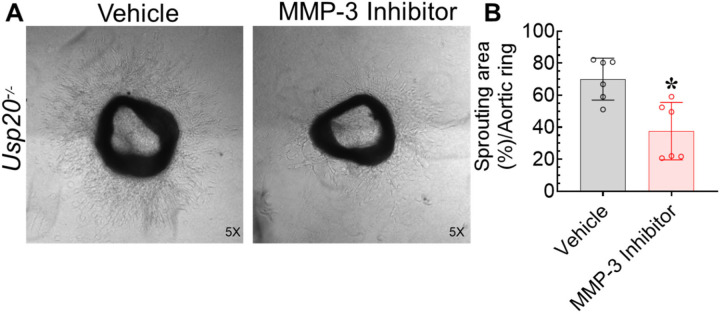
Aortic sprouting in *Usp20*^*−/−*^ samples is attenuated by MMP3 inhibition. (A) Representative micrographs of the *ex vivo* aortic ring sprouting assay with 1 μM MMP3 inhibitor treatment using the aorta obtained from 3-month-old *Usp20*^−/−^ male mice. (B) Quantification of the sprouting area stated in panel A. Magnification 5x. N = 2 male *Usp20*^−/−^ mice; each mouse represents six dots (3 untreated and three treated with MMP3 inhibitor); each dot represents the average sprouting area of 7 aortic rings. Each bar represents the mean ± SD. *p < 0.05 vs vehicle, Student-t test.

Since MMP3 was increased in sprouts from both *Usp20*^*−/−*^ and USP20 S334D mice (Fig. 8), and endothelial MMP3 expression is regulated by NFκB^28^ we further focused on this protein identified in our proteome profiling assay. To test the proangiogenic role MMP3 in USP20-modulated angiogenesis, we performed the aortic ring assay using the aorta from the *Usp20*^−/−^ mice and treated them with vehicle or MMP3 inhibitor II, (1μM). In our sprouting assays, MMP3 inhibitor significantly reduced angiogenesis as compared with sprouting in vehicle-treated samples (Fig. 9). This suggests that USP20 regulates MMP3 production by attenuating NFκB signaling, and thus acts as a brake in the process of angiogenesis.

## DISCUSSION

Our findings demonstrate a novel role for USP20 activity and its Ser334 phosphorylation in EC sprouting and angiogenic growth in the vasculature. A major signaling axis in this process is ubiquitination-driven NFκB signaling, leading to gene transcription of MMP3 amidst a handful of pro-angiogenic factors. Thus, a reduction in USP20 expression and activity or an increase in USP20 S334 phosphorylation as seen in human atherogenic samples^6,7^ can engender an increase in NFκB signaling, as well as angiogenic growth leading to neo vessel formation in an atherosclerotic plaque. Neovascularization in a healthy artery is required to nourish the adventitia and outer medial layer because the vasa vasorum-derived microvessels are not penetrating beyond these layers. However, in a pathological condition like atherosclerosis, arterial wall thickness increases along with an increased proliferation of the vasa vasorum and intimal neovascularization^29^. Accumulating evidence suggests that angiogenesis in the atherosclerotic plaque is more aggressive and destabilizes the plaque^30,31^. Histological analysis of the atherosclerotic plaques revealed that microvessel density is positively correlated with macrophage infiltration, lipid-rich lesions and thin-cap atheroma formation^29^. Accordingly, increased neovascularization in the atherosclerotic plaque could promote vulnerability of atherosclerotic plaque and lead to adverse vascular remodeling.

Endothelial dysfunction is a key event in early atherosclerosis and is induced by NFκB-mediated transcription of TNF and IL-6^32^. Elevated NFκB activity in the atherosclerotic lesion plays a pleiotropic role at different stages of atherogenesis by upregulating the transcription of several proinflammatory genes, including TNF, IL-1β, IL-6, MCP-1, and ICAM-1^33^. Activation of NFκB signaling induces proliferation, migration, and phenotypic switching of the VSMCs, leading to matrix accumulation and cap formation in the atherosclerotic plaque^34^. Microvessels expressing NFκB-inducing kinase (NIK), a component of the noncanonical NFκB pathway, were more prevalent in unstable atherosclerotic plaques^25^. These NIK-positive microvessels correlated significantly with CD34-positive endothelial cells, suggesting a link between NFκB activation and increased neovascularization in atherosclerotic lesions^25^. Therefore, inhibiting NFκB activity has been regarded as a promising approach to attenuate the progression of atherosclerosis^35^, and might be applicable to other diseases driven by NFκB signaling such as cancers^36–38^ and retinopathy^39^.

EC migration is a critical step in pathological angiogenesis during neovascularization^40^ and proinflammatory cytokines increase both EC migration and proliferation. Proinflammatory cytokine-induced activation of NFκB signaling is associated with the expression of adhesion molecules on ECs and subsequent transmigration of leukocytes^41^. Our findings confirm that NFκB signaling is required for EC migration (Figure 5A–B) and this is also affected by USP20 expression and S334 phosphorylation. Our previous work demonstrated that USP20 reverses lysine-63 ubiquitination of the signaling adaptor TRAF6 and suppresses proatherogenic stimuli-induced NFκB activation in mouse VSMCs and subsequently attenuates atherosclerosis and neointimal hyperplasia^5–7^. In this current study, we demonstrate that USP20 suppresses proinflammatory cytokine-induced NFκB activation in primary ECs, as confirmed by an increase in phosphorylation of p65 in IL-1β-stimulated *Usp20*^*−/−*^ ECs versus WT ECs (Figure 1). NFκB activity was augmented in MCECs with overexpression of USP20-DN but diminished with exogenous expression of USP20 WT (Figure 2). Concordantly, our *ex vivo* aortic ring sprouting assay revealed increased angiogenic sprouting in *Usp20*^*−/−*^ mice as compared with WT littermates (Figure 6). Data from our *in vitro* spheroid assay also demonstrated that MCECs transduced with USP20-DN adenovirus increased angiogenic sprouting compared to the GFP control, whereas MCECs transduced with USP20-WT adenovirus decreased angiogenic sprouting (Figure 4). Furthermore, inhibition of NFκB signaling with TPCA-1 significantly decreased angiogenic sprouting in aorta as well as in MCEC spheroids (Figures 6 & 7). These findings underscore the important role of USP20 in suppressing angiogenesis by downregulating NFκB activity in ECs.

The association of USP20 with its substrate is regulated by its phosphorylation status at a specific Ser residue (Ser333 in human USP20 and Ser334 in murine USP20). We recently showed that USP20 phosphorylation is significantly elevated in SMCs from human atherosclerotic segments compared with SMCs from nonatherosclerotic segments^7^. This earlier study further demonstrated that USP20-S334A mice developed ~50% less neointimal hyperplasia than the congenic WT mice after carotid endothelial denudation^7^. Additionally, isolated SMCs from the USP20-S334A mice stimulated significantly lesser NFκB activation and cell proliferation than WT SMCs^7^. We now demonstrate a novel role for USP20 Serine334 phosphorylation in angiogenesis. Furthermore, our findings suggest that, the phospho-mimetic USP20 S334D is both pro-inflammatory and angiogenic; whereas the phospho-defective USP20 S334A is anti-inflammatory and perhaps angiostatic.

Our proteome profiler analyses identified MMP3^42^ a transcriptional target gene of NFκB signaling as an angiogenic factor regulated by USP20. Absence of USP20 (*Usp20*^*−/−*^) and expression of USP20 S334D engender a marked increase in NFκB signaling, as well as an increase in MMP3 gene expression in aortic angiogenesis. MMP3 is a proangiogenic factor and provokes an increase in VEGF in healthy adults^43^. Inflammation-induced upregulation of MMP3 is mediated via NFκB activation in rabbit aortic and human saphenous vein VSMC^44^. In a rodent model of pulp injury induction, MMP3 mRNA expression and protein levels significantly increased in the ECs after 24 hours post injury. A topical application of MMP3 on the injured pulp tissue of rats significantly increased angiogenesis after 24 hours of treatment^45 46^. The extracellular matrix associated with atherosclerotic plaques is susceptible to MMP3-mediated degradation leading to plaque destabilization^47^. While MMP3 is regarded as a marker for atherosclerotic plaque vulnerability, and as a prognostic marker in ischemic heart disease, studies also imply that an evaluation of MMP3 genetic polymorphisms is needed to correlate risk versus disease outcomes^42^.

Our findings support an important role for MMP3 in angiogenesis driven by NFκB signaling that is regulated by USP20 activity, since the inhibition of MMP3 significantly decreased angiogenic sprouting in the *Usp20*^*−/−*^ aortic rings. Taken together, our studies suggest that EC USP20 negatively regulates angiogenesis by attenuating NFκB signaling and its downstream target, MMP3. Molecules or compounds that activate USP20 can serve as novel therapeutics to mitigate plaque growth in atherosclerosis, and could be useful as angiostatic compounds to prevent tumor growth.

## Supplementary Material

Supplement 1

Supplementary Materials:

This article includes Supplementary Figures S1–S4

## Data Availability

All data needed to evaluate the conclusions in the paper are present in the paper or the Supplementary Materials.
