# Supplementary material for: Inflammation-induced endothelial cell activation and angiogenic sprouting are downmodulated by ubiquitin-specific peptidase 20
Source: bioRxiv. 2025 May 24:2025.05.20.655129. Preprint. [Version 1] doi: 10.1101/2025.05.20.655129 (PMC12139994; doi:10.1101/2025.05.20.655129)
Supplement: Supplement 1 [file media-1.pdf]

Inflammation-induced endothelial cell activation and angiogenic sprouting are downmodulated by ubiquitin-specific peptidase 20

Bipradas Roy[1], Jiao-hui Wu[1], Neil J. Freedman[1, 2] & Sudha K. Shenoy[1, 2]

[1] Division of Cardiology, Department of Medicine, Duke University Medical Center, Durham, NC 27710, USA

[2]Department of Cell Biology, Duke University Medical Center, Durham, NC 27710, USA

*Address correspondence to skshenoy@dm.duke.edu

Supplementary Figures

Figure S1

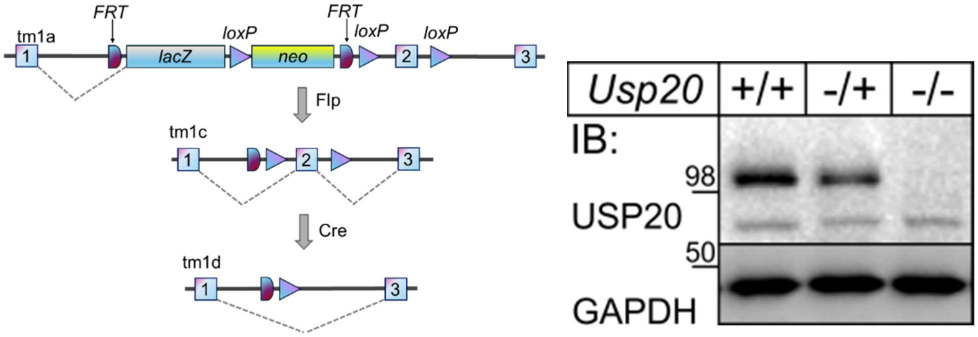

Figure S1. **Usp20(-/-) mouse generation.** Schematic shows the targeting vector design that generates knockout first allele and the breeding steps that produce Usp20(-/-). The schematic is adapted from a model presented by KOMP and EUCOMM Breeding Strategies [13]. Western blots show cardiac lysates of C57BL/6-congenic mice of the indicated genotype that were immunoblotted serially for USP20 and GAPDH (as a loading control). Shown are results from a single experiment, representative of 3 performed with distinct mice of each genotype.

Figure S2

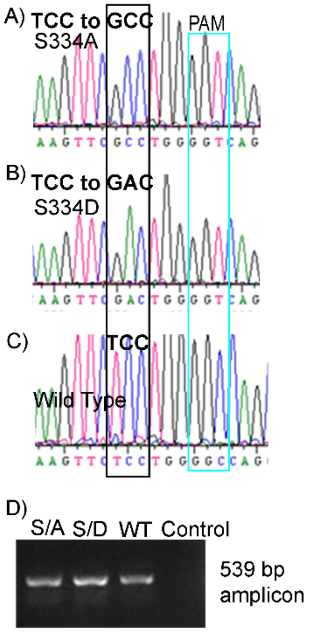

Figure S2. **USP20 S334A and S334D CRISPR/CAS9 knock-in mice.** Panels **A** and **B** show sequence chromatograms of USP20 mutations: TCC to GCC, (Ser to Ala), TCC to GAC (Ser to Asp) confirmed by founder analysis and allelic subcloning. Panel **C** shows the WT sequence TCC (Ser334). Panel **A** and **B** also show the intended silent mutation (GGC to GGT) in the repair oligo to disrupt Protospacer Adjacent Motif (PAM) sequence. **D**) DNA gel showing the 539 bp amplicon of USP20 exon 9 region that contains the targeted site Ser334.

Figure S3

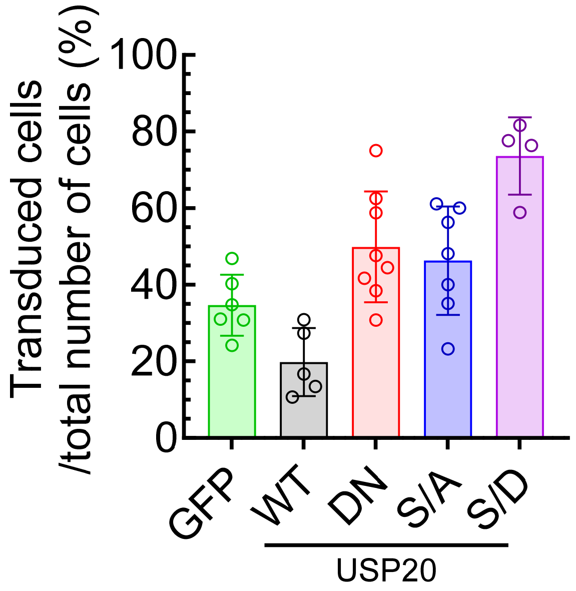

Figure S3. Transduction efficiency of recombinant adenoviruses encoding N-terminal HA-tagged USP20 constructs in MCECs. A portion of cells prepared for experiments summarized in Fig 3 were set aside to conduct imaging shown in Fig 3C. The bar graph is this panel summarizes the counts of HA-USP20 positive cells normalized by total number of cells that are DAPI positive.

Figure S4

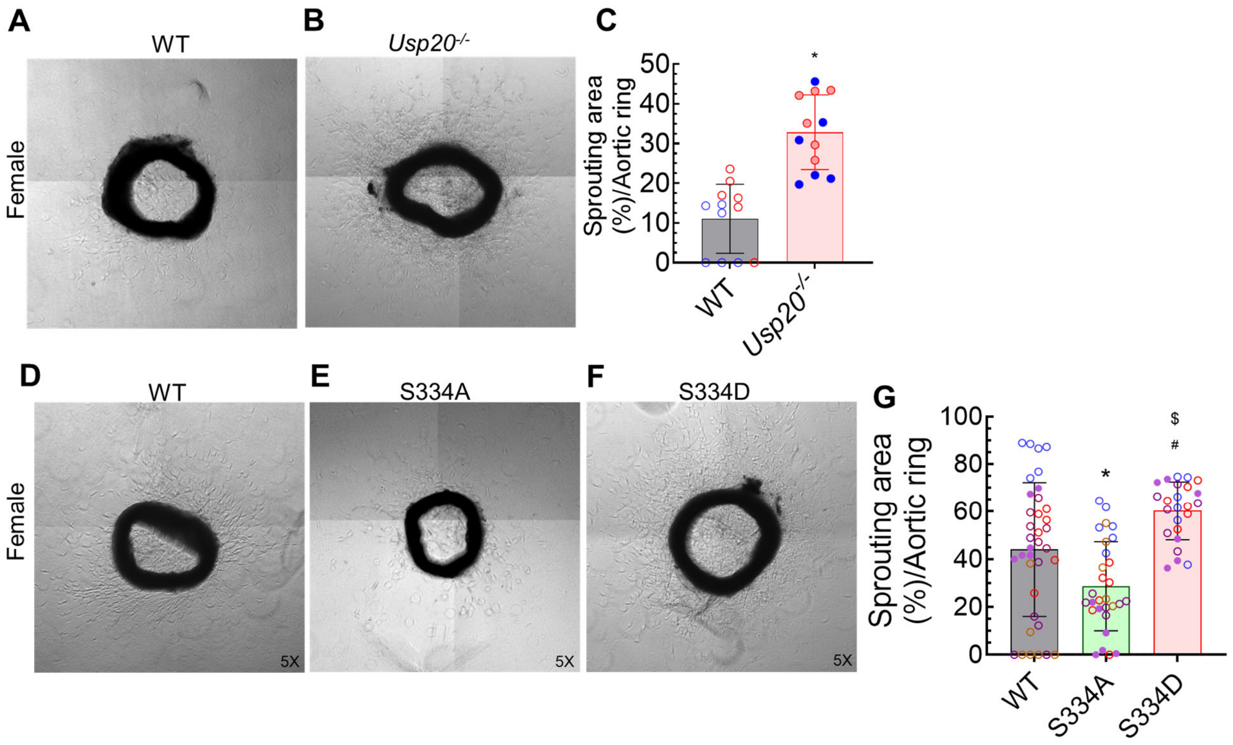

Figure S4. Effect of USP20 and Ser334 phosphorylation on aortic angiogenesis. (A-B) Representative micrographs of aortic sections isolated from 3-month-old WT and *Usp20*$^{-/-}$ female mice. (C) Quantification of the sprouting area of WT and *USsp20*$^{-/-}$ samples from A-B. Magnification 5x. N=2 mice for each group; each mouse represents 6 dots; each dot represents the average sprouting area of 4-7 aortic rings. Each bar represents the mean ± SD. An unpaired student's t-test was performed to determine the statistical significance. *$p < 0.01$ vs WT. (D-F) Representative micrographs of the *ex vivo* aortic ring sprouting assay using the aorta isolated from 3-month-old WT, USP20-S334A, and Usp20-S334D female mice, respectively. (G) Quantification of the sprouting area stated in panels K-M. Magnification 5x. N= 6 WT, 3 USP20-S334A, and 3 USP20-S334D mice; each mouse represents 6 dots; each dot represents the average sprouting area of 5-7 aortic rings. Each bar represents the mean ± SD. One-way ANOVA and Holm-Šídák's multiple comparisons tests were performed to determine the statistical significance. *$p < 0.05$ vs WT-CTL, $^{\#}p < 0.01$ vs USP20-S334A as indicated. WT, Wild-Type.
